# The prevalence and incidence of pharmacologically treated diabetes among older people receiving home care services in Norway 2009–2014: a nationwide longitudinal study

**DOI:** 10.1186/s12902-022-01068-6

**Published:** 2022-06-14

**Authors:** Tonje Teigland, Jannicke Igland, Grethe S. Tell, Johannes Haltbakk, Marit Graue, Anne-Siri Fismen, Kåre I. Birkeland, Truls Østbye, Mark Peyrot, Marjolein M. Iversen

**Affiliations:** 1 Department of Health and Caring Sciences, Western Norway University of Applied Sciences, Bergen, Norway; 2 Department of Global Public Health and Primary Care, University of Bergen, Bergen, Norway; 3 Institute of Clinical Medicine, University of Oslo, Oslo, Norway; 4 Department of Transplantation Medicine, Oslo University Hospital, Oslo, Norway; 5 Department of Family Medicine and Community Health, Duke University, Durham, NC USA; 6 Department of Sociology, Loyola University Maryland, Baltimore, MD USA

**Keywords:** Pharmacologically treated diabetes, Home care services, Older people, Prevalence, Incidence, Time trends

## Abstract

**Background:**

A substantial proportion of older people who receive home care services (HCS) has diabetes and requires diabetes specific monitoring, treatment and self-care assistance. However, the prevalence and incidence rates of diabetes among older people in HCS are poorly described. The aim of the study is to estimate prevalence, incidence and time trends of pharmacologically treated diabetes among older people receiving HCS in Norway 2009–2014.

**Methods:**

This nationwide observational cohort study is based on data from two population registries. The study population consisted of persons registered in the Norwegian Information System for the Nursing and Care Sector aged ≥ 65 years receiving HCS during at least one of the years 2009–2014. The Norwegian Prescription Database was utilized to identify participants’ prescriptions for glucose lowering drugs (GLD). The period prevalence was calculated each year as persons with one or more prescriptions of GLD in the current or previous year. Incident cases were defined as subjects receiving prescriptions of GLD for the first time in the given calendar year if there were no prescriptions of any GLD for that person during the previous two years.

**Results:**

From 2009 to 2014, the number of older people receiving HCS increased from 112,487 to 125,593. The proportion of these who received GLD increased from 14.2% to 15.7% (*p* < 0.001) and was significantly higher among men than women. The annual incidence rate of diabetes among those receiving HCS showed a decreasing trend from 95.4 to 87.5 cases per 10,000 person-years from 2011 to 2014, but when stratifying on age group and gender, was significant only among the oldest women (age groups 85–89 years and 90 +).

**Conclusions:**

The increasing prevalence of older people with diabetes who receive HCS highlights the importance of attention to treatment and care related to diabetes in the HCS.

**Supplementary information:**

The online version contains supplementary material available at 10.1186/s12902-022-01068-6.

## Background

There has been a shift in the epidemiology of diabetes in old age, and worldwide persons aged 65–79 years has the highest prevalence of diabetes [1, 2]. In Norway, it is estimated that approximately 245,000 people (4.7%) have diabetes [3]. The use of blood glucose-lowering drugs (GLD) increased in the general population from 2005 to 2011 [4]. The prevalence of pharmacologically treated diabetes in 2011 was 3.2%, while the incidence rate was 313 per 100,000 person years. There was a peak in prevalence at approximately 78 years, while there was a trend of decreasing incidence of users of GLD in the older segment of the general population [4]. Further, during 2009–2014, in the general Norwegian population aged 30–89 years the incidence of pharmacologically and non-pharmacologically treated type 2 diabetes decreased (from 609 to 398 cases per 100,000 person years), while the prevalence continued to rise and increased with age [5].

As the population of older people is growing it is expected that the demand for home care services (HCS) will increase [6]. The Norwegian government provides health care coverage for all residents, this also encompasses HCS which is managed and financed by the local municipalities [7]. All residents in Norway may apply to their local municipality to receive HCS. Common reasons to apply for HCS are altered self-care capacity due to illness, recovery after a hospital stay, impaired health, or age-related functional decline. The care contracted by the municipalities is defined by an evaluation of each applicant’s functional level, and the amount of care is defined as number of days or number of hours per period. In most cases, a person in need of public healthcare, will primarily be offered HCS so that he/she can stay at home as long as possible before moving to a nursing home [7]. In 2012 The Norwegian Coordination Reform was implemented, aiming to reduce the overall specialist health care cost and to improve the coordination between primary and specialist care [8, 9]. More responsibility for health care services was relocated to primary care due to the reform, increasing the workload on HCS.

As a substantial amount of care is contracted HCS, allocating resources should have high priority to provide adequate services when self-management ability of older people with diabetes is suboptimal. However, there has been limited research on the prevalence and incidence of diabetes in HCS (pharmacologically and non-pharmacologically treated). Previous studies investigating the prevalence of diabetes among persons receiving HCS have been small cross-sectional studies in various healthcare settings, which are often hampered by selection bias. The reported diabetes prevalence has been 20%–30% [10–14]; none of these studies reported diabetes incidence rates or include trends in prevalence and incidence over time.

Both ageing and diabetes are risk factors for functional decline and disability [1] and can generate a need for HCS. To our knowledge, large population-based studies have not been conducted to determine the prevalence and incidence of diabetes among people receiving HCS. In order to allocate resources necessary to provide adequate amount and high-quality services to maintain adequate monitoring, treatment and self-care assistance, there is a need for trend estimations of prevalence and incidence of diabetes in HCS. Therefore, the aim of this study was to estimate prevalence and incidence of pharmacologically treated diabetes, including time trends, in the population of older people receiving HCS in Norway from 2009 to 2014.

## Methods

### Study design

This nationwide observational study is based on data from two population registries: the Norwegian Information System for the Nursing and Care Sector (IPLOS) and the Norwegian Prescription Database (NorPD), during 2009–2014. The two registries were merged by Statistics Norway (SSB) utilizing the personal identification number unique to each Norwegian resident. IPLOS contains data about persons who have had contracted HCS [15]. NorPD contains data on dispensed drugs from all out-patient pharmacies in Norway [16].

### Study population and definitions

Information on all persons in Norway aged ≥ 65 years receiving HCS from 1 January 2009 to 31 December 2014 was obtained from IPLOS. Information on the amount of contracted and received time of service and living situation was retrieved. We excluded persons receiving only a limited amount of HCS (< 14 h or < 14 separate days in a given calendar year) as they may not be representative of the general population of HCS recipients. These cutoff points were chosen because they represented the 5^th^ percentile for the distribution of both days of care and hours of care. Data from the NorPD identifies persons with pharmacologically treated diabetes. Codes from The Anatomical Therapeutic Chemical (ATC) system in NorPD were used to identify persons with pharmacologically treated diabetes by filled prescriptions with ATC code A10A (insulins and analogues) and ATC code A10B (blood glucose lowering drugs, excl. insulins) [17]. In addition, information on year of birth and sex was obtained from SSB. Furthermore, for simplicity, we use the term diabetes to describe pharmacologically treated diabetes in this article. Thus, persons with diet-treated diabetes are grouped together with persons without diabetes, and for simplicity referred to as not having diabetes. Due to insufficient information about diagnoses in IPLOS, it was not possible to estimate the prevalence of non-pharmacologically treated diabetes.

**Fig. 1:**
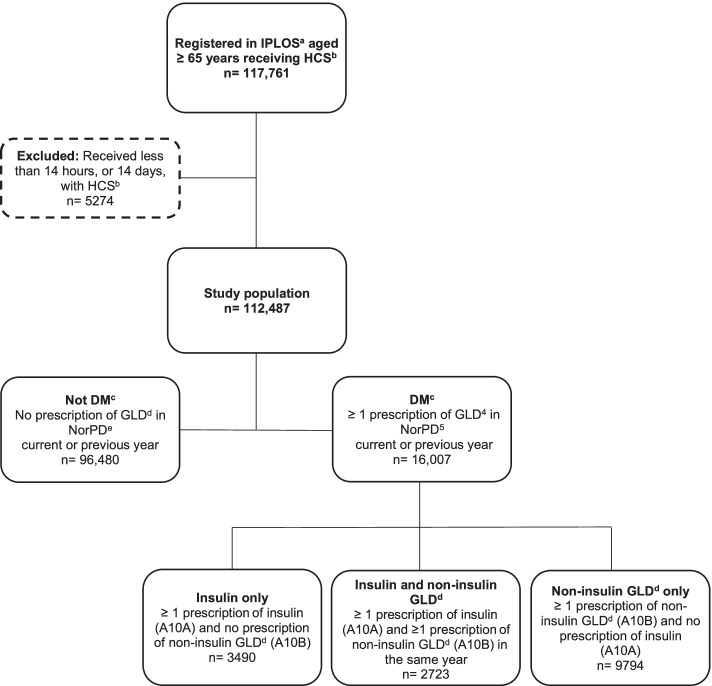
Study population recruited from IPLOS^a^ and linked to data from NorPD^e^, showing data from 2009. ^a^ IPLOS = the Norwegian Information System for the Nursing and Care Sector, ^b^ HCS = Home care service, ^c^ DM = Diabetes mellitus, pharmacologically treated, ^d^ GLD = Glucose lowering drugs, ^e^ NorPD = Norwegian Prescription Database. Prescriptions with ATC code A10A (insulins and analogues) is defined as insulin, prescriptions with ATC code A10B (blood glucose lowering drugs, exclusive insulins) is defined as non-insulin glucose lowering drugs (non-insulin GLD) and prescriptions with either ATC code A10A or A10B is defined as glucose lowering drugs (GLD)

The flowchart of the study population is shown in Fig. 1 using prevalence numbers from 2009. Corresponding study populations were defined for each of the years 2010–2014.

### Measures of diabetes occurrence

For both prevalence and incidence, filled prescriptions with either ATC code A10A (insulins and analogues) or A10B (blood glucose lowering drugs, excluding insulins) were defined as GLD. Prescriptions with ATC code A10A (insulins and analogues) were defined as insulin, and prescriptions with ATC code A10B (blood glucose lowering drugs, excluding insulins) were defined as non-insulin GLD. These definitions identified four subgroups: persons prescribed insulin only; persons prescribed non-insulin GLD only; persons prescribed both insulin and non-insulin GLD; and persons not prescribed GLD, defined as not having diabetes (Fig. 1).

Prevalence outcome measures:

- Individuals with ≥ 1 prescription of GLD in the current or previous year were defined as prevalent users.
- Prevalent users of “Insulin only” had at least one prescription with insulin and no prescription of non-insulin GLD during the current and previous year.
- Prevalent users of “Non-insulin GLD only” had at least one prescription of non-insulin GLD and no prescription of insulin the current and previous year.
- Prevalent users of “Insulin and non-insulin GLD” had at least one prescription of both insulin and non-insulin GLD the current or previous year, with both types of treatments prescribed during the same calendar year.
- In calculation of prevalence proportion, the denominator was the total number of persons receiving HCS by sex, age group and calendar year.

Incidence outcome measures: Individuals prescribed ≥ 1 GLD in the current year, and no such prescription during the previous 24 months, were defined as incident users. Incident numbers are not available for 2009 and 2010, as no wash-out period was available for these years, so incidence was analyzed only for 2011–2014. Flowchart of the study cohort illustrating definition of incident users is shown in Additional file 2: Supplementary Fig. 1. In calculating diabetes incidence rates, each person contributed one person-year in the denominator for a given calendar year if he/she had received at least 14 days or 14 h of HCS and with no prescription of GLD the previous two calendar years. Example: A person who received HCS in 2011, 2012 and 2013 and had the first prescription with GLD in 2012, contributed one (non-incident) person-year in 2011 and one (incident) person-year in 2012, but none in 2013 and 2014.

### Statistics

Descriptive statistics, t-tests and chi-square tests were used to describe the study population and to investigate differences between those with and without registered diabetes. Log-binominal regression with calendar year included as a continuous covariate, linear regression, ordinal logistic regression and quantile regression was used to test for time trends in characteristics (age, gender, living situation and hours of received care). Log-binominal regression with calendar year included as a continuous covariate was also used to test for log-linear trends in prevalence, and Poisson regression with calendar year included as a continuous covariate to test for log-linear trends in incidence. The reported risk ratios (RR) from log-binomial regression and incidence rate ratios (IRR) from negative binomial regression can be interpreted as relative change per calendar year, assuming a log-linear trend. All regression models were stratified by sex and age group and adjusted for age within each age group (except for the group 90 + years, where we lack information on exact age due to data restrictions [privacy policies]). The only variable containing missing values was living situation. Missing values for this variable was handled with listwise deletion when testing for trend in Table 1. The variable was not used in further analyses in the manuscript. All analyses were performed in STATA version 16. A significance level of *p* < 0.05 was used in all analyses.

**Table 1:** Characteristics of persons aged ≥ 65 years receiving home care services^a^ in Norway

Variable	**2009**	**2010**	**2011**	**2012**	**2013**	**2014**	***p***^***b***^
N	112,487	117,673	119,307	122,566	123,674	125,593	
Women, n (%)	74,391 (66.1)	77,387 (65.8)	78,258 (65.6)	79,809 (65.1)	79,902 (64.6)	80,676 (64.2)	< 0.001
Age, mean (SD)	82.0 (6.9)	82.0 (7.1)	82.0 (7.2)	81.9 (7.3)	81.8 (7.4)	81.8 (7.5)	< 0.001
Age group, n (%)							
65–69	8159 (7.3)	9083 (7.7)	9963 (8.4)	10,883 (8.9)	11,669 (9.4)	11,910 (9.5)	0.010^c^
70–74	10,476 (9.3)	11,057 (9.4)	11,238 (9.4)	11,871 (9.7)	12,339 (10.0)	13,197 (10.5)	
75–79	17,226 (15.3)	17,251 (14.7)	17,201 (14.4)	17,242 (14.1)	17,469 (14.1)	17,706 (14.1)	
80–84	26,618 (23.7)	27,247 (23.2)	27,074 (22.7)	27,500 (22.4)	26,533 (21.5)	26,393 (21.0)	
85–89	30,681 (27.3)	31,121 (26.5)	30,768 (25.8)	30,539 (24.9)	30,252 (24.5)	30,254 (24.1)	
90 +	19,327 (17.2)	21,914 (18.6)	23,063 (19.3)	24,531 (20.0)	25,412 (20.6)	26,133 (20.8)	
Living alone^d^, n (%)	62,764 (62.7)	67,448 (63.8)	69,615 (63.7)	70,542 (63.1)	72,413 (62.9)	73,289 (62.7)	0.003
DM^e^, n (%)	16,007 (14.2)	17,985 (15.3)	18,393 (15.4)	18,839 (15.4)	19,291 (15.6)	19,752 (15.7)	< 0.001
Hours of HCS^f^, median (IQR)	52 (14–161)	52 (15–162)	52 (17–168)	52 (16–167)	53 (16–167)	52 (15–165)	0.99

^a^ Excluding the lowest 5th percentile, defined as receiving < 14 days or 14 h of home care services during at least one of the years 2009–2014

^b^ Binominal regression with calendar year included as a continuous covariate, linear regression, ordinal logistic regression and quantile regression was used to test for trends

^c^ p-value reflect test of overall difference

^d^ Missing: *n* = 12,416 in 2009, *n* = 11,963 in 2010, *n* = 9936 in 2011, *n* = 10,771 i 2012, *n* = 8509 in 2013 and *n* = 8616 in 2014

^e^ DM = Diabetes mellitus, defined as a person registered in the The Norwegian Prescription Database (NorPD) with at least one prescription of insulins and analogues (A10A) or blood glucose lowering drugs, excl. insulin (A10B) in the current or previous year

^f^ HCS = Home care services

## Results

### Characteristics

During 2009–2014 the number of persons receiving HCS in Norway increased by ~ 12%, from 112,487 in 2009 to 125,593 in 2014 (Table 1). The majority (~ 65%) was women and the mean age was relatively stable during the study period (~ 82 years). In all study years, the largest proportion of persons were in the age group 80–89 years. During the study period, the proportion of persons in age groups 65–69, 70–74 and 90 + years increased. Median number of hours of HCS per year was stable at 52 from 2009–2014. Approximately 63% of the participants were living alone, and among them ~ 26% were men.

Compared to those without diabetes, those with diabetes were younger and a smaller proportion was living alone in both 2009 and 2014 (Table 2). There was a higher proportion of men in the group with diabetes, compared to the group without diabetes (40% vs 33%, respectively). Further, compared to the group without diabetes, the group with diabetes received more hours of HCS both in 2009 and 2014. Those with diabetes received a median of 62 (IQR 18–187) hours of HCS per year, while those without received 52 (IQR 14–160) hours of HCS per year in 2014.

**Table 2:** Description of the study population, with and without pharmacologically treated diabetes, in 2009 and 2014

Variable	**Not DM**^**a**^ **2009**	**DM**^**a**^ **2009**	***p***^***b***^	**Not DM**^**a**^ **2014**	**DM**^**a**^ **2014**	***p***^***b***^
N	96,480	16,007		10,5841	19,752	
Women, n (%)	64,807 (67.2)	9584 (59.9)	< 0.001	69,707 (65.9)	10,969 (55.5)	< 0.001
Age, mean (SD)	82.3 (6.9)	80.5 (7.0)	< 0.001	82.1 (7.4)	79.9 (7.4)	< 0.001
Age group n (%)						
65–69	6674 (6.9)	1485 (9.3)	< 0.001^c^	9481 (9.0)	2429 (12.3)	< 0.001^c^
70–74	8556 (8.9)	1920 (12.0)		10,425 (9.9)	2772 (14.0)	
75–79	14,163 (14.7)	3063 (19.1)		14,196 (13.4)	3510 (17.8)	
80–84	22,481 (23.3)	4137 (25.8)		21,770 (20.6)	4623 (23.4)	
85–89	26,913 (27.9)	3768 (23.5)		26,239 (24.8)	4015 (20.3)	
90 +	17,693 (18.3)	1634 (10.2)		23,730 (22.4)	2403 (12.2)	
Living alone, n (%)	54,314 (63.4)	8450 (58.9)	< 0.001	62,664 (63.6)	10,625 (57.5)	< 0.001
Hours of HCS^d^, median (IQR)	52 (13–157)	60 (18–189)	< 0.001	52 (14–160)	62 (18–187)	< 0.001

^a^ DM = Diabetes mellitus, defined as a person registered in the The Norwegian Prescription Database (NorPD) with at least one prescription of Insulins and analogues (A10A) or Blood glucose lowering drugs, excl. insulin (A10B) in the current or previous year

^b^ T–tests, chi-square tests and median test are used to investigate the difference between those with and without pharmacologically treated diabetes in 2009 and in 2014, respectively

^c^ p-value reflect test of overall difference

^d^ HCS = Home care services

### Prevalence of pharmacologically treated diabetes

**Fig. 2:**
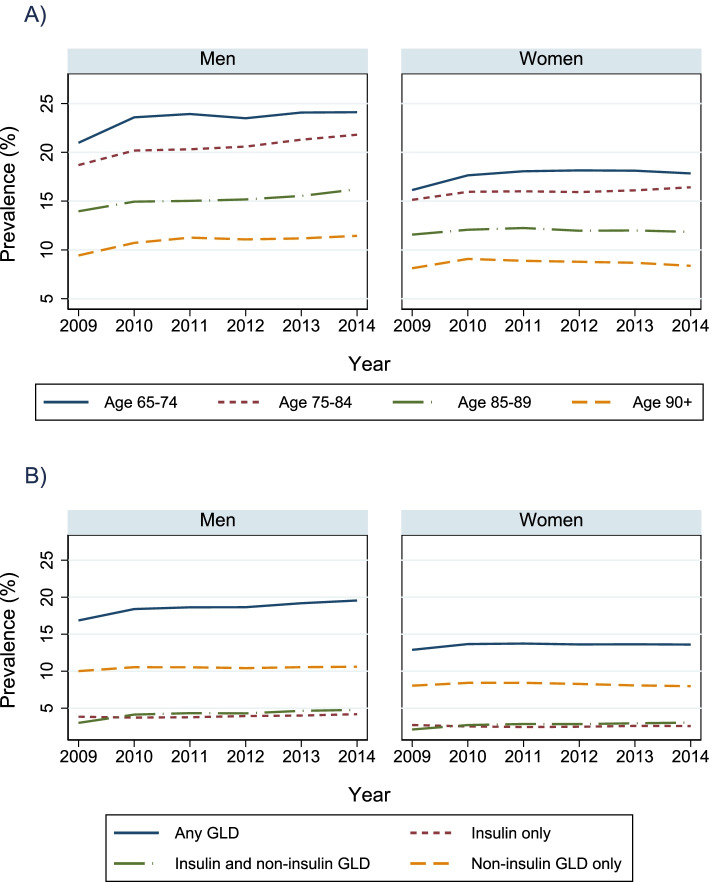
Time trends in prevalence of pharmacologically treated diabetes among persons receiving home care services^a^. Data presented in panel **A** is stratified by age group and gender, in panel **B** by type of treatment and gender. ^a ^Excluding the lowest 5th percentile, defined as receiving < 14 days or 14 h of home care services during at least one of the years 2009–2014. GLD = glucose lowering drugs. Prescriptions with ATC code A10A (insulins and analogues) is defined as insulin, prescriptions with ATC code A10B (blood glucose lowering drugs, exclusive insulins) is defined as non-insulin glucose lowering drugs (non-insulin GLD) and prescriptions with either ATC code A10A or A10B is defined as glucose lowering drugs (GLD)

The overall prevalence of diabetes in the HCS population increased from 14.2% in 2009 to 15.7% in 2014 (Table 1). In all years and all age groups, diabetes in HCS was more prevalent in men than women (*p* < 0.01) (Fig. 2A). Both women and men had a significant increase in prevalence over time (Additional file 1: Supplementary Table 1). Prevalence of diabetes increased for men in all age groups and for women in age groups 65–74 and 75–84 years (Fig. 2A and Additional file 1: Supplementary Table 2).

In men, there was an increase in persons treated with “Insulin only” and “Insulin and non-insulin GLD”, while the prevalence of those treated with “Non-insulin GLD only” was stable. The proportion of women using both “Insulin and non-insulin GLD” increased, those treated with “Non-insulin GLD only” decreased. The proportion of women treated with “Insulin only” did not change (Fig. 2B and Additional file 1: Supplementary Table 1). Among all persons with pharmacologically treated diabetes (labeled “any GLD” in Fig. 2B), 17% used both “Insulin and non-insulin GLD” in 2009 vs. 23.3% in 2014.

### Incidence of pharmacologically treated diabetes

**Fig. 3:**
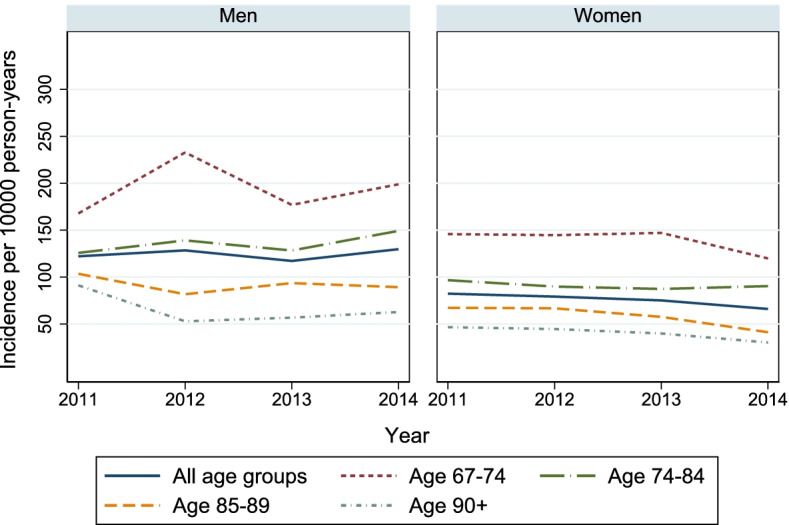
Time trends in incidence of pharmacologically treated diabetes among persons receiving home care services^a^. Data presented is stratified by age group and gender. ^a^ Excluding the lowest 5th percentile, defined as receiving < 14 days or 14 h of home care services during at least one of the years 2009–2014

The overall crude incidence rate of diabetes per 10,000 person-years decreased from 95.4 in 2011 to 87.5 in 2014. After adjustment for age and gender there was an average reduction of 4% which was significant (IRR (95% CI): 0.96 (0.93–0.99)). When stratifying on gender incidence of diabetes showed a significant decrease among women from 2011 to 2014, on average 7% per year. Further stratification on age group showed a significant reduction only in the age groups 85 years and older (Fig. 3 and Additional file 1: Supplementary Table 3). The trend was not significant among men.

## Discussion

This is the first study to estimate the prevalence and incidence of pharmacologically treated diabetes in a nationwide population of older persons receiving HCS. From 2009 to 2014 the total number of HCS recipients 65 years and older in Norway increased by about 12% and the prevalence of diabetes increased from 14.2% to 15.7%. There was increased prevalence of diabetes among men and the youngest women and a decreased incidence of diabetes among the oldest women.

Among recipients of HCS in Norway the overall crude prevalence of diabetes increased modestly from 14.2 to 15.7% during 2009–2014. To our knowledge, trends in prevalence and incidence of diabetes in HCS have not been reported previously. Other studies have reported higher prevalence of diabetes in HCS; however, these studies also included non-pharmacologically treated diabetes, which would yield higher estimates, and/or were based on self-report, which can bias results. Differences in age distribution between studies may also explain different results. Additionally, it is difficult to compare results across countries and studies as criteria for admission into HCS differ among countries [6]. In some countries informal care (given by spouse, children etc.) may be more common than formal care [6]. Despite different care models, our findings may nevertheless be relevant to other populations of elderly with diabetes with care needs in their diabetes self-management.

The increasing prevalence of diabetes in HCS from 2009 to 2014 is consistent with estimates in the Norwegian general population [4, 5]. However, in the study by Ruiz et al. [5] only patients with type 2 diabetes were included. In our HCS population the prevalence was higher, probably reflecting that the HCS population is sicker than the population as a whole [18]. In older adults with pharmacologically treated diabetes, special care is required in monitoring pharmacologic therapies when older peoples’ self-management ability is suboptimal [19]. Worldwide the trend of increasing diabetes prevalence among older people is expected to continue, resulting in increased public health and economic challenges [2].

We found that age and sex-adjusted incidence rates of diabetes decreased during 2009–2014, but when stratifying on gender and age group the rates were stable, except for a decreasing trend among women aged 85 years or older. In contrast, previous studies in the Norwegian general population aged 70 years or older detected trends towards declining incidence in use of oral antidiabetic drugs (in the period 2005–2011) and of type 2 diabetes (in the period 2009–2014) in both men and women [4, 5]. One possible explanation is that recipients of HCS who are in regular contact with health care personnel are more likely to have their symptoms of diabetes detected earlier than in the general population of older people. Another possibility is that HCS recipients have a higher risk of developing type 2 diabetes. The incidence rates might also be influenced by introduction of HbA1c as diagnostic criterion, which was implemented in Norwegian guidelines in 2012. Ruiz et al. [5] found in their analyses of data from the general population a small reduction in rate of change in incidence after this. Further follow-up will be required in order to establish long-term trends in prevalence and incidence of diabetes in HCS.

The proportion of older people with type 1 diabetes is increasing worldwide due to increasing incidence and survival rates, and it is expected that this group will require closer attention with regards to diabetes care in the future [20]. Although we did not try to classify subjects according to diabetes type, the majority of participants that use “Insulin only” as glucose lowering treatment most likely have type 1 diabetes, even if some older people with type 2 diabetes might also use “Insulin only” (ex. in case of kidney failure) [4]. We observed an increased prevalence among men in this group. An increasing prevalence of type 1 diabetes in HCS would demand competence and resources able to meet the attention this group requires, e.g. in term of preventing hypoglycemic events and providing individualized treatment as described by Schütt et al. [20].

There was an increasing prevalence of persons treated with the combination of “Insulin and non-insulin GLD” in women and men. Among those with diabetes, 17% used both “Insulin and non-insulin GLD” in 2009 vs. 23.3% in 2014. Norwegian guidelines from 2009 recommend that persons with type 2 diabetes in need of GLD, should start treatment with Metformin, and if not reaching treatment goals, should add insulin or another non-insulin GLD [21]. Hence, an increase in the percentage of persons treated with “Insulin and non-insulin GLD” might reflect an increase in persons with type 2 diabetes not reaching treatment goals (in general, HbA1c > 53.0 mmol/mol (7%)) with non-insulin GLD only. The large increase in people using “Insulin and non-insulin GLD” indicates that more people are utilizing more complex treatment and care than previously.

We found that in HCS the youngest age groups (65–74 and 75–84 years) had the highest proportions of persons with diabetes. Additionally, in these age groups both women and men had an increasing prevalence while the incidence was stable. Longevity for those with diabetes and continued reception of HCS represents a challenge for health care providers in HCS. Health care provider expertise is required when self-management ability is suboptimal and/or for those with more advanced diabetes complications [19]. In addition to the increasing prevalence proportion, there has been an increase in the number of persons receiving HCS. This might be a consequence of the aging of the general population. However, the national coordination reform may also have increased the number of older persons receiving HCS in Norway [8]. This increase in HCS recipients results in an even higher increase in the number of persons with diabetes in the HCS. Thus, if the trends of both an increasing number of persons receiving HCS and an increasing prevalence of diabetes in HCS continue, the demand for resources necessary to provide adequate quality of HCS to older persons with diabetes will also increase [22, 23].

The major strength of this study is the use of data from nationwide registries, covering all residents receiving HCS and all outpatient pharmacy prescriptions [24]. Thus, this study presents a valid and reliable calculation of national prevalence of those with pharmacologically treated diabetes in HCS. For incidence outcome measures we used a “wash-out period” of 24 months, due to the possibility that a person could have been prescribed GLD for more than 12 months use. This reduces the possibility of misclassification of prevalent cases as incident. However, this study also has some limitations. Due to inadequate recording of diabetes diagnosis in IPLOS it was not possible to estimate the prevalence of non-pharmacologically treated diabetes or the total diabetes prevalence as the sum of pharmacologically and non-pharmacologically treated diabetes [25]. Information on type of diabetes also was unavailable. We have information on the number of hours of HCS received, but were unable to verify that the care delivered was related to diabetes specifically, as the IPLOS registry does not have information on why people receive HCS. Many of these elderly individuals may have several conditions and comorbidities, and the overall functional level is the determinant for how many hours of HCS is offered. The main value of our current study of prevalence and incidence is to provide a base for calculating the need for diabetes related services, but effectiveness of such services is an important question for future research. Another limitation is the lack of information about sociodemographic characteristics such as education and ethnicity, which was unavailable due to regulations regarding personal data protection. However, among persons 70 years and older in Norway there were only 4% foreign born in 2016 [26].

## Conclusion

In Conclusion, despite a modest decrease in the incidence of diabetes among receivers of HCS, the prevalence increased from 2009 to 2014. Although other recipients of HCS also have disabilities, functional decline and diseases with complex comorbidities, the increasing prevalence of diabetes will undoubtedly place a higher demand on HCS. As special care is required for older people with pharmacologically treated diabetes, consequences can be expected in terms of increased demands on required resources and needed expertise among health care providers delivering this care.

## Supplementary information

**Additional file 1.**

**Additional file 2.**

## Data Availability

The datasets generated and/or analysed during the current study are not publicly available due to data protection regulations. Personal data protection legislation and the approval from the Regional Ethical Committee prohibits data sharing for the purpose of reproducing the results. Researchers can apply for ethical approval and obtain data from the registry holders (service@helsedata.no).

## Abbreviations

ATC: Anatomical Therapeutic Chemical
DM: Diabetes mellitus
HCS: Home care services
GLD: Glucose lowering drugs
IPLOS: The Norwegian Information System for the Nursing and Care Sector
IRR: Incidence rate ratios
NorPD: The Norwegian Prescription Database
RR: Risk ratios
SSB: Statistics Norway
