# Correction: The prevalence and incidence of pharmacologically treated diabetes among older people receiving home care services in Norway 2009–2014: a nationwide longitudinal study

**DOI:** 10.1186/s12902-022-01091-7

**Published:** 2022-07-14

**Authors:** Tonje Teigland, Jannicke Igland, Grethe S. Tell, Johannes Haltbakk, Marit Graue, Anne-Siri Fismen, Kåre I. Birkeland, Truls Østbye, Mark Peyrot, Marjolein M. Iversen

**Affiliations:** 1 Department of Health and Caring Sciences, Western Norway University of Applied Sciences, Bergen, Norway; 2 Department of Global Public Health and Primary Care, University of Bergen, Bergen, Norway; 3 Institute of Clinical Medicine, University of Oslo, Oslo, Norway; 4 Department of Transplantation Medicine, Oslo University Hospital, Oslo, Norway; 5 Department of Family Medicine and Community Health, Duke University, Durham, NC USA; 6 Department of Sociology, Loyola University Maryland, Baltimore, MD USA

**Correction: BMC Endocr Disord 22, 159 (2022)**

**https://doi.org/10.1186/s12902-022-01068-6**

After publication of this article [1], it was brought to our attention that the Fig. 1 is incorrect, the correct Fig. 1 is shown below:

**Fig. 1:**
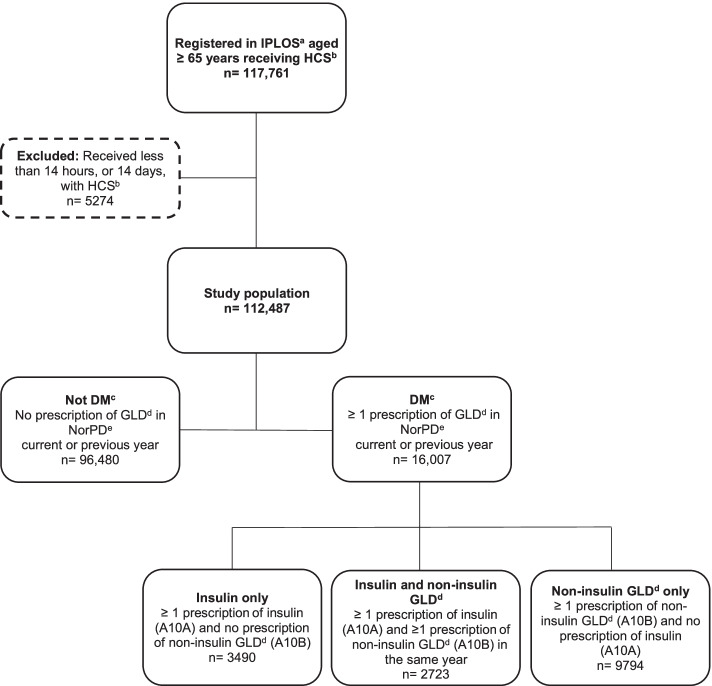
Study population recruited from IPLOS^a^ and linked to data from NorPD^e^, showing data from 2009. ^a^ IPLOS = the Norwegian Information System for the Nursing and Care Sector, ^b^ HCS = Home care service, ^c^ DM = Diabetes mellitus, pharmacologically treated, ^d^ GLD = Glucose lowering drugs, ^e^ NorPD = Norwegian Prescription Database. Prescriptions with ATC code A10A (insulins and analogues) is defined as insulin, prescriptions with ATC code A10B (blood glucose lowering drugs, exclusive insulins) is defined as non-insulin glucose lowering drugs (non-insulin GLD) and prescriptions with either ATC code A10A or A10B is defined as glucose lowering drugs (GLD)

The original publication has been corrected.
