# Supplementary material for: The prevalence and incidence of pharmacologically treated diabetes among older people receiving home care services in Norway 2009–2014: a nationwide longitudinal study
Source: BMC Endocr Disord. 2022 Jun 14;22:159. doi: 10.1186/s12902-022-01068-6 (PMC9195364; doi:10.1186/s12902-022-01068-6)
Supplement: Supplementary file 1 — Additional file 1. [file 12902_2022_1068_MOESM1_ESM.docx]

|  | **Risk ratio** | **CI** | ***p*** |
| --- | --- | --- | --- |
| Men, |  |  |  |
| Any GLD | **1.02** | **1.02-1.03** | **<0.001** |
| Insulin only | **1.02** | **1.00-1.03** | **0.010** |
| Non-insulin GLD only | 1.01 | 1.00-1.01 | 0.107 |
| Insulin and non-insulin GLD | **1.06** | **1.05-1.08** | **<0.001** |
| Women, |  |  |  |
| Any GLD | **1.01** | **1.00-1.01** | **0.020** |
| Insulin only | 0.99 | 0.98-1.00 | 0.227 |
| Non-insulin GLD only | **0.99** | **0.99-1.00** | **0.032** |
| Insulin and non-insulin GLD | **1.05** | **1.04-1.06** | **<0.001** |

**Additional file 1**

**Supplementary Table 1: Test for trend in prevalence of diabetes in different treatment groups in home care services in Norway 2009-2014, stratified by gender**

Log-binominal regression with calendar year included as a continuous covariate were used to test for trends in prevalence. The regression models were stratified on sex and adjusted for age.

**Supplementary Table 2: Test for trend in prevalence of pharmacologically treated diabetes in home care services in Norway 2009-2014, stratified by sex and age groups**

|  | **Risk ratio** | **CI** | ***p*** |
| --- | --- | --- | --- |
| Men, |  |  |  |
| 65-74 years | **1.02** | **1.01-1.03** | **<0.001** |
| 75-84 years | **1.03** | **1.02-1.04** | **<0.001** |
| 85-89 years | **1.03** | **1.01-1.04** | **<0.001** |
| 90+ years | **1.03** | **1.01-1.05** | **0.009** |
| Women, |  |  |  |
| 65-74 years | **1.02** | **1.01-1.03** | **0.003** |
| 75-84 years | **1.01** | **1.00-1.02** | **0.001** |
| 85-89 years | 1.00 | 0.99-1.01 | 0.684 |
| 90+ years | 0.99 | 0.98-1.01 | 0.788 |

Log-binominal regression with calendar year included as a continuous covariate were used to test for trends in prevalence. The regression model were stratified on sex and age groups and adjusted for age within each age group.

**Supplementary Table 3: Test for trend in incidence of pharmacologically treated diabetes in home care services in Norway 2011-2014, stratified by sex and age groups**

|  | **IRR** | **CI** | ***p*** |
| --- | --- | --- | --- |
| Men, | 1.00 | 0.96-1.05 | 0.86 |
| 67-74 years | 1.01 | 0.94-1.09 | 0.72 |
| 75-84 years | 1.04 | 0.98-1.11 | 0.202 |
| 85-89 years | 0.97 | 0.87-1.07 | 0.527 |
| 90+ years | 0.89 | 0.67-1.04 | 0.132 |
| Women, | 0.93 | 0.89-0.96 | <0.001 |
| 67-74 years | 0.94 | 0.87-1.02 | 0.146 |
| 75-84 years | 0.98 | 0.92-1.04 | 0.42 |
| 85-89 years | **0.86** | **0.79-0.93** | **<0.001** |
| 90+ years | **0.87** | **0.79-0.97** | **0.011** |

Poisson regression with calendar year included as a continuous covariate were used to test for trends in incidence. The regression models were stratified on sex and age groups and adjusted for age within each age group.
