# Supplementary material for: The prevalence and incidence of pharmacologically treated diabetes among older people receiving home care services in Norway 2009–2014: a nationwide longitudinal study
Source: BMC Endocr Disord. 2022 Jun 14;22:159. doi: 10.1186/s12902-022-01068-6 (PMC9195364; doi:10.1186/s12902-022-01068-6)
Supplement: Supplementary file 2 — Additional file 2. [file 12902_2022_1068_MOESM2_ESM.docx]

**Additional file 2**


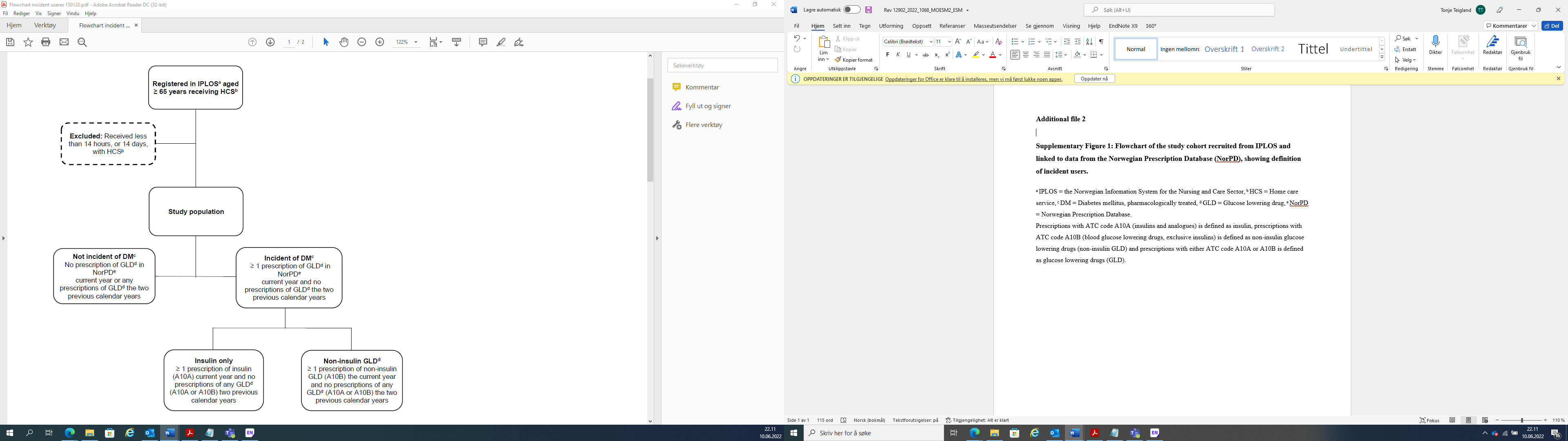


**Supplementary Figure 1: Flowchart of the study cohort recruited from IPLOS and linked to data from the Norwegian Prescription Database (NorPD), showing definition of incident users.**

^a^ IPLOS = the Norwegian Information System for the Nursing and Care Sector, ^b^ HCS = Home care service, ^c^ DM = Diabetes mellitus, pharmacologically treated, ^d^ GLD = Glucose lowering drug, ^e^ NorPD = Norwegian Prescription Database.

Prescriptions with ATC code A10A (insulins and analogues) is defined as insulin, prescriptions with ATC code A10B (blood glucose lowering drugs, exclusive insulins) is defined as non-insulin glucose lowering drugs (non-insulin GLD) and prescriptions with either ATC code A10A or A10B is defined as glucose lowering drugs (GLD).
